# Qualitative investigation of barriers to accessing care by people who inject drugs in Saskatoon, Canada: perspectives of service providers

**DOI:** 10.1186/1747-597X-8-35

**Published:** 2013-10-01

**Authors:** Katherine Lang, Jaycie Neil, Judith Wright, Colleen Anne Dell, Shawna Berenbaum, Anas El-Aneed

**Affiliations:** 1 Department of Pharmacy Services, Regina Qu’Appelle Health Region, 2180 - 23rd Avenue, Regina, SK S4S 0A5, Canada; 2 College of Pharmacy and Nutrition, University of Saskatchewan, Thorvaldson Building, 110 Science Place, Saskatoon, SK S7N 5C9, Canada; 3 Public Health Services, Saskatoon Health Region, Saskatoon City Hospital 701 Queen Street, Saskatoon, SK S7K 0M7, Canada; 4 Department of Sociology & School of Public Health, University of Saskatchewan, 9 Campus Drive, Saskatoon, SK S7N 5A5, Canada

**Keywords:** People who inject drugs (PWID), Addiction, Service providers, Qualitative research, Focus groups

## Abstract

**Background:**

People who inject drugs (PWID) often encounter barriers when attempting to access health care and social services. In our previous study conducted to identify barriers to accessing care from the perspective of PWIDs in Saskatoon, Canada: poverty, lack of personal support, discrimination, and poor knowledge and coordination of service providers among other key barriers were identified. The purpose of the present investigation was to explore what service providers perceive to be the greatest barriers for PWIDs to receive optimal care. This study is an exploratory investigation with a purpose to enrich the literature and to guide community action.

**Methods:**

Data were collected through focus groups with service providers in Saskatoon. Four focus groups were held with a total of 27 service providers. Data were transcribed and qualitative analysis was performed. As a result, concepts were identified and combined into major themes.

**Results:**

Four barriers to care were identified by service providers: inefficient use of resources, stigma and discrimination, inadequate education and the unique and demanding nature of PWIDs. Participants also identified many successful services.

**Conclusion:**

The results from this investigation suggest poor utilization of resources, lack of continuing education of health care providers on addictions and coping skills with such demanding population, and social stigma and disparity. We recommend improvements in resource utilization through, for example, case management. In addition, sensitivity training and more comprehensive service centers designed to meet PWID’s complex needs may improve care. However, community-wide commitment to addressing injection drug issues will also be required for lasting solutions.

## Background

People who inject drugs (PWIDs) experience excess mortality when compared to the general population, [1,2] and are at risk of developing serious health conditions such as blood-borne disease, psychiatric illness, bacterial infections, and overdose [3,4]. Despite poor health, PWIDs do not seek care as often as people who do not inject drugs [5-7]. Many studies have examined causes for underutilization of health care from the perspective of PWIDs, identifying issues ranging from system deficiencies to personal factors such as PWID favouring the use of addictive substances over seeking medical help [8-14]. PWIDs cite poor interactions with service providers as a main reason for avoiding care and have reported discrimination from a variety of service providers [11,15-17]. In addition, PWIDs maintain that some service providers are unable to provide effective services because service providers lack education regarding issues surrounding injection drug use [9,11,16,18].

Service providers’ experiences while treating PWIDs and their opinions regarding this population have also been examined. Discriminatory attitudes are prevalent among service providers and can be due to difficult behaviours exhibited by PWIDs. In addition, it is reported that service providers discriminate against people with blood-borne diseases [17,19], a co-morbidity with PWIDs. In general, greater exposure to PWIDs can improve service providers’ attitudes toward this population, and those that specialize in areas such as infectious disease have more positive views of PWIDs [20,21]. Service providers and PWIDs have similar views regarding the lack of education among service providers. Many investigations have found that service providers are uncomfortable when providing treatment to PWIDs [22,23], and have trouble navigating available services [10,24]. Some providers are unaware that injecting drugs is a problem in their community [10]. In addition, some service providers exhibit differing views regarding the ethical and legal aspects of harm reduction programming [24-26] or may fear that their business/clinical practice will suffer if they serve PWIDs [25,26]. Furthermore, service providers in previous studies have also cited lack of system resources as a barrier to providing the best possible care to PWIDs. These include limited programs and services, poor access to services and extensive paperwork [10,24,27].

While the opinions of service providers regarding PWIDs have been investigated, limited studies exist that primarily focus on barriers to accessing care by such a vulnerable population. The literature provides limited guidance regarding the opinions of service providers, providing care to PWIDs, in a small urban setting regarding barriers to accessing care [10].

In 2009, we conducted a study in the urban centre of Saskatoon, Canada, (population 223 200) to examine the experiences of PWIDs attempting to access care during a crisis [14]. Since 2009, Saskatoon Health Region has seen the highest rate of HIV in Canada, an epidemic driven primarily by injection drug use [28,29]. The PWID focus group participants indicated that they encountered many barriers when attempting to find care, including lack of system resources, discrimination, lack of communication with service organizations, insufficient financial resources, and poor social support networks [14]. Recommendations suggested in the study included peer leadership programmes, such as, training PWIDs to disseminate information, condoms and needles to their peers. However, such recommendations cannot be implemented until we gain information from the other side of the process; the service providers.

As this research was driven by a community coalition including service providers, it was necessary to collect information that was suitable to advise future policy and programming for PWIDs in the community. Through consultation with community members, it was determined that the opinions of service providers should be taken into consideration before recommending any new policy/programs to address these barriers, as service providers would be actively involved in the mobilization of any potential changes. As discussed earlier, limited published studies exist that are applicable to Saskatoon. There were twelve studies we were able to partially compare our work to, but they were not as closely related; the focus of these studies (in comparison to our work) is shown in Table 1.

**Table 1:** Comparable studies to our work

**# of studies**	**Focus of the study in comparison to our work**	**Citation**	**Date**
3	Methadone maintenance therapy	[30-32]	2007, 2007, 1997
1	Aboriginal populations only	[33]	2010
1	Needle exchange programs	[28]	2012
1	Naloxone for opiate overdose	[34]	2006
1	Harm minimization	[35]	2005
1	General drug and alcohol use	[22]	1991

Literature searches were done on the following databases: Medline, PubMed, Embase, PsycInfo, ProQuest Public Health and Google Scholar. The search terms allied/health occupations, service providers, physicians assistants, intravenous substance abuse, substance-related disorders, attitude of health personnel, health services accessibility and health care delivery were used. The details of the search strategy used and the references for the comparable studies are shown in Additional file 1.

Among the available studies, stigmatization and a lack of education/training were prevailing themes found in the literature [10,22,24,30,33,34,36]. Other barriers identified in the literature include waiting lists [8,37], limited hours and financial resources [27,31,33], lack of confidentiality/privacy [31,36] as well as a concern for service providers’ personal safety [35]. In addition, the literature showed that a lack of health insurance was an important barrier, either due to a lack of eligibility or due to the extensive process that must be completed. Such a barrier is mostly applicable within a jurisdiction lacking universal health care [8,24].

Our study is an exploratory investigation with a purpose to enrich the literature and to guide community action. It provides global perspectives for service providers who work with PWIDs without being profession-specific. However, the collected data will guide future research in which we may focus on specific health provider group(s). We attempted to explore the overall experience of service providers while caring for PWIDs during a crisis in an urban centre, in order to maintain and improve service delivery. As identified by participants in our first phase, crises for PWIDs can include homelessness, physical trauma and drug-related problems, such as overdose or exhausting their drug supply [14]. In this study we examined the opinions of service providers regarding barriers for PWIDs to access care.

## Methods

### Community collective [BRIDGE Saskatoon]

This work was undertaken through the partnership of the University of Saskatchewan, the Saskatoon Health Region, and the community collective BRIDGE (Building Relationships around Injection Drug Use for Greater Engagement) Saskatoon. BRIDGE Saskatoon consists of service providers, researchers, community members, and other key stakeholders who have an interest in the welfare of PWIDs in Saskatoon. The purpose of this collective is to address health promotion and primary prevention, harm reduction, enforcement, and treatment and recovery in a collaborative fashion [38]. The information from both the first and second phase of this investigation will guide the members of the collective in improving services for those who inject drugs in Saskatoon.

### Research framework

Qualitative methodology was deemed to be the most appropriate data collection method for the purpose of this investigation. Focus groups were utilized to allow participants to provide observations, as well as expand on these observations providing a better understanding of the topic [32]. Group dynamics present in a focus group setting provide additional depth to the information collected, as they allow for the participants to further reflect on the opinions of others [39]. The use of focus groups is advantageous as it allows participants to direct the conversation [32], ensuring that the values and issues important to the participants supersede those of the researchers. However, focus groups have limitations as well. A focus group session can be skewed by a few dominant group members and participants may feel pressured to give similar answers to each other. As well, focus groups are not fully confidential or anonymous and with the sensitive nature of injection drug use, participants may not express their true views.

Ethical approval to conduct this research was granted by the University of Saskatchewan Behavoural Ethics Board (number 10–142).

### Sample

The sample strategy was purposeful and convenient. Possible agencies for recruitment were identified through our previous focus groups with PWIDs, in which services used by PWIDs in Saskatoon were identified [14]. The researchers contacted these organizations via phone and/or email for prospective participants. Verbal announcements of the prospective focus groups were also made at local injection drug use educational events to recruit participants. In addition, members of BRIDGE Saskatoon were asked to circulate a recruitment email among their personal contacts. In order to be included in the study, service providers were required to have provided services to PWIDs in the last two years. Twenty-seven service providers were recruited for the study; the sample included physicians (n = 3), nurses (n = 7), pharmacists (n = 2), counsellors (n = 6), outreach workers and social workers (n = 4) and providers who have a management role (oversee the operation of the organization) (n = 5). We recognize the limited sample size and diversity of service providers as a limitation. However, this was an exploratory study; based on which, future work will be conducted. In this study, we did not probe for profession-specific beliefs. Our intent was rather to provide an overview assessment of the beliefs and attitudes of health care providers in general within addiction services.

### Data collection and instruments

Data was collected during the months of August and September of 2010. Four focus groups were conducted, with the number of participants ranging from three to ten people. Allocation of members to focus groups was arbitrary, based on convenience for participants. Written consent was obtained by each participant prior to the focus groups. Discussions were facilitated by the principal investigator, as well as two research assistants who received training from the principal investigator regarding focus group moderation and facilitation. All groups were audio-taped with the permission of the participants, and transcribed by a member of the research team. Participants were served lunch, and no honorarium was provided.

The discussion guide was designed to complement the questions asked in our recent study with PWIDs in Saskatoon [14]. The research team developed the question guide, then presented it to members of BRIDGE Saskatoon (which includes service providers who work with PWIDs) for revisions and guidance. Five questions were developed that were complementary to the questions asked to PWIDs:

• What has been your experience with injection drug users (IDUs*) who access services during a health crisis?**

• Did you ever, on behalf of IDU clients, try to navigate other services (the system) and, if so, what was the outcome?

• When providing services to IDU clients do you or other health staff at your facility interact with IDU patients differently than other patients. If yes, how?

• Do you feel you have the training you need to provide the best service possible to IDU clients?

• What are some of the resources that are helpful when providing a service to an IDU client?

*The term Injection Drug User (IDU) was used by BRIDGE and was adopted by Saskatoon based service providers. However, in this manuscript, we used the widely accepted term People Who Inject Drugs (PWIDs).

**Although this is how the question was phrased, a crisis was further described by focus group moderators as encompassing health and social crises.

### Data analysis

All audio-tapes were transcribed by a research team member. An inductive qualitative data analysis process involving reading, coding, displaying, reducing and interpreting was then used. The focus was on content analysis – primarily the determination of themes arising from the transcripts. The researcher began with immersion – reading and rereading texts and reviewing notes. Broad emerging themes were identified and codes attached. Each thematic area was then explored and information reduced to its essential points. At each step, core meanings from the text were interpreted. Finally, an overall interpretation of the study findings was made with a focus on showing how thematic areas related to one another, explaining how the themes related to our original research questions, and suggesting what these findings may mean beyond the specific context of our study.

## Results

Four major themes emerged from the focus group discussions. Participants shared their thoughts about discrimination towards PWIDs, and commented about the state of their education regarding injection drug use. They noted deficiencies and inefficiencies in the use of system resources, and explained that PWIDs can be a complex and unique population to interact with. This is due to PWIDs’ behaviour, their addiction and their complex psychological and physical condition. Service providers also acknowledged that there are excellent services for PWIDs in the city, and described the traits of individuals and organizations that make these services so successful. It should be noted that barriers, identified in this study, can either be PWID related such as behavioural, or service provider related such as discrimination and prejudice.

### Theme 1: discrimination

There were varying views regarding discrimination of PWIDs among the participants in this investigation. It was mentioned that discrimination occurs at many levels, not just from service providers to the PWIDs, but also from the community towards the PWIDs and among the service providers themselves. However, some indicated that within their organization, PWIDs are treated the same as everyone else. Some emphasized that when a person who injects drugs is medically sick, and not sick due to their drug abuse, their treatment is equal to those who do not inject drugs. The focus group facilitators defined discrimination as interacting with people who inject drugs differently than other patients. Discrimination is the result of multiple factors including ethnicity, or drug use [15,40]. Identifying the precise source of discrimination was not in the scope of this study. It was not specifically probed and it is difficult to determine the source of discrimination described by the focus group participants.

#### ***Service providers towards PWIDs***

The majority of participants had witnessed discrimination among their colleagues, due to factors such as ethnicity, the presence of communicable disease, or simply the fact that the clients inject drugs. Participants in our study described how PWIDs have avoided utilizing the most appropriate care (for example, primary care or minor emergency services) due to discrimination, or avoided care altogether until the health problem is acute. Although most participants witnessed discrimination in other services providers, some did describe treating people who inject drugs differently themselves. In both situations, it was acknowledged that the *behaviour* associated with addiction was the cause for providers to become frustrated with PWIDs, rather than the fact that they inject drugs.

“It’s not because you use drugs. That’s really not, my first thought is . . . it’s more like, this is disruptive, they’re yelling at my staff, they’re being rude to my other customers . . . it’s not that they’re an injection drug user, it’s the behaviour that you’re reacting too.”

It is not surprising that service providers react negatively to the behaviour of PWIDs since it is established that there is a linkage between mental health and drug use [28]. The mental health issues are possibly one explanation of the poor behaviour.

In addition, some service providers felt they had to advocate for people who inject drugs because of the discriminatory attitudes they experienced with their colleagues.

“. . . so it’s hard to advocate for services and I’ve tried to phone for somebody who is suicidal and as soon as the psychiatrist heard that they had a history of cocaine use he didn’t want to see them at all . . . he considered it a complete waste of his time. So I had to find another route. And that’s unfortunately some of the advocacy and other work that has to be done in order to find the right match.”

#### ***Community towards PWIDs***

Participants also noted that the general public was a major source of discrimination, and explained that there is little support from the community or the government for providing services to this population as well as limited financial support. They described negative comments about services for PWIDs in the local media, particularly needle exchange programs and expressed the view that the government does not prioritize funding for PWIDs and related services. They also discussed how community members do not want services for PWIDs in their area:

“We had a representative from the . . . community association come up to the [needle exchange] van and she was all fired up and she said, [the community is] just sick and tired of seeing all these clients out in the community . . . and there’s needles all over the place and we’re trying to get some of the higher up politicians on board to shut you guys down.”

Although some community members do not support harm reduction services for PWIDs, such as the needle exchange van, our previous work indicated that the van was identified by PWIDs as a much needed and successful service in Saskatoon [14]. Without the support of the community and the government it makes it difficult to secure the finances to expand and improve on these services. Also, it can be expected that PWIDs are less likely to use services such as harm reduction when there is such a negative public view surrounding it.

#### ***Providers towards PWID service providers***

Not only can discrimination be a barrier for PWIDs to access services, but it can also become a barrier between service providers. The literature suggests that caring for patients with certain conditions (such as HIV/AIDS) is associated with perceived societal stigma. In a study conducted in China, perceived societal stigma resulted in a higher level of negative impact on patients [38]. In this study, one service provider observed that stigma may be directed towards service providers who care for PWIDs. This may lead to service providers not providing optimal care to a person who injects drugs in fear of how they will be perceived by their colleagues and the public.

“And I think as people who work with . . . that population, you can see it come towards you as that [you] support [that] person . . . You get a sense or a feeling [of], “why do you think this person matters? Why do you work with this individual . . .” Or if their behaviours are a little unmanageable, they’re looking at you like you’re the parent, and so . . . you should take care of that.”

### Theme 2: education

Almost all service providers agreed that they are not provided with enough information about injection drug use during their formal education. Participants were confident when providing the medical aspects of a PWID’s care, but felt uncomfortable when dealing with the social issues that can accompany addiction. Medical service providers can be faced with a PWID’s addictions, social instability and other comorbidities associated with drug use. Physicians and nurses rarely receive training regarding effective strategies for managing such difficulties frequently encountered with people who inject drugs [41].

“And I don’t think that’s been addressed through education of professionals. How to actually . . . care for these individuals . . . The average nurse is probably getting close to [age] 50. Well, as a person of that age, we didn’t receive any education on addictions.”

Furthermore, some described a basic or scientific understanding of injection drug use, but recognized that they do not understand the concepts of how drugs are used in the community:

“And I don’t even know . . . if someone says well yeah . . . I do a gram of coke – is that a lot, is that not very much, is that . . . in a day?”

The participants agreed that one of the most challenging aspects of caring for PWIDs was providing effective and expedited referral between needed services. They explained that identifying services that could address the diverse needs of PWIDs was extremely difficult, and indicated that they did not know where to find this type of information. It was suggested that a point person or directory would be useful for this purpose.

“So when someone approaches you and says, I have this issue, whether I’m single or . . . got three kids, you can go through this one stop shopping . . . when they’re in crisis, we take them when they’re in withdrawal, we look after their kids.”

The participants made reference to some excellent formalized educational opportunities, such as methadone days. These days allow service providers to learn about basic methadone pharmacology, opioid dependency counseling, mental comorbidity and many other topics regarding methadone therapy. However, some reported using their own time to learn about injection drug use. Nevertheless, most conceded that the best education came from experience, good mentoring, and listening to the PWIDs themselves. In addition, the participants described varying levels of willingness to learn about injection drug use among themselves and their colleagues. Some service providers, either when speaking for themselves or for their colleagues, did not want to learn, or found that the area is too complex to attempt to understand. However, many other providers expressed a willingness to learn, and some participants observed that education about injection drug use could decrease discrimination and stigma.

“It’s not just stigma, it could be just lack of understanding . . . really what recovery is . . . and what that looks like for people [who inject drugs] and . . . ignorance contributes to stigma and discrimination.”

### Theme 3: inefficient use of limited resources

#### ***Lack of basic resources***

One of the greatest barriers that service providers encountered when attempting to provide care was insufficient resources. The participants discussed unacceptable wait lists, and indicated that a lag time between accessing services and provision of appropriate care could cause clients to lose interest in seeking treatment. They also explained that many services, such as methadone maintenance therapy or psychiatric counselling, had to be delayed or cancelled due to staffing and workload issues.

“So the big thing is wait lists within our own system. Like we have two clients who will be waiting about six months for extra counselling which I think is one of the biggest barriers. They need help now, six months down the road, we’re probably going to lose them, they’re not going to want to go through with it.”

The participants noted that most services lacked funding, physical space, or sufficient hours of operation to be useful to clients. Some service providers in our study expressed frustration that basic resources, such as housing services or affordable transportation, were in short supply, which hindered their ability to help their clients.

“Housing, there’s just a lack of housing, there’s a lack of supports. Yes, we can, we don’t have a barrier connecting them with outreach, that’s a very quick thing, but from beyond that we can’t just make housing appear.”

#### ***Limited services, disjointed services, and lack of cooperation***

In addition, service providers observed that limited access to some resources could lead to inefficient use of other resources. For example, the participants had seen PWIDs utilize limited detoxification beds for shelter, or use the emergency room for non-emergent situations.

“Having worked in [the emergency room], where you have people coming with heart attacks, you have kids coming in with asthma attacks, you have people coming in with fresh strokes, the person who’s coming in withdrawing is going to wait . . . because if you, going to triage somebody withdrawing with an abscess versus a heart attack, you are going to triage that heart attack. You have to!”

Participants also indicated that their clients made progress in facilities such as addiction treatment centers or detention facilities, but explained that PWIDs reverted back to their old behaviours when placed back in the community without continuing support. They further observed that services are physically spread out, making it hard for PWIDs to attend all of their appointments. The service providers explained that although they may find it difficult to refer the clients to suitable services, it is even more difficult for PWIDs to know where to go to address their many and complex needs. However, service providers agreed that it would be ideal for services in the city to address all the needs of PWIDs in one place.

“. . . some of the services are like . . . I can give you a bed, but I can give you no programming to teach you life skills. So you have a bed, but you still don’t know how to cook, you don’t know how to take care of yourself . . .”

Participants recognized that the people who work within the system can also contribute to its inefficiency. They described the negative effect that crossing boundaries could have. Rather than working collaboratively, service providers are more concerned about who will be responsible for a particular patient and want to avoid “stepping on the toes” of other providers. They also explained that their own colleagues could create barriers when attempting to find care for PWIDs.

“. . . And you got personal care homes but they are a private business, so they can pick and choose who they want and then that’s where the addictive behaviour becomes the barrier.”

#### ***Policy impact***

While staffing and service locations/coordination were blamed for inefficient use of resources, regulatory policies also contribute to this issue. Participants indicated that certain aspects of care, such as methadone dispensing, the associated rules, and regulations could be inhibiting.

“. . . with the methadone . . . there’s a separate sheet, there’s times that are tracked, there has to be double staff . . . it’s just really complicated and it . . . puts more pressure on us as staff.”

PWIDs in the first phase of the study also reported restrictive aspects of policy, including those policies of methadone and detoxification [14].

### Theme 4: working with a unique and demanding population

The participants recognized that unlike regular patients, working with PWIDs may require additional efforts. They explained that PWIDs can be taxing simply because interactions can be demanding and time consuming. Service providers discussed that PWIDs can be impatient, rude, and disruptive, and that this behaviour can exhaust an already time-pressured service provider. Many participants observed that PWIDs can be manipulative, and further explained that this can lead to poor medical care as service providers don’t have accurate medical information about the patient.

“I think it’s hard to differentiate . . . when are you getting manipulated, and when are they truly in pain . . . Are you really in pain, or are you just trying to get drugs.”

Furthermore, the participants noted that PWIDs generally have more complex and unique health needs than other clients. They explained that PWIDs frequently get medically sick, which sometimes only becomes apparent when drug use has decreased or stopped. Participants discussed the issues of long hospital stays, hyperacute pain response, dental problems, and the consequences of blood-borne infections such as human immunodeficiency virus (HIV) and hepatitis C virus (HCV). Service providers explained how mental health issues can complicate the care and treatment of substance users, and noted the large effect of this “dual diagnosis” on PWIDs. This is not surprising since it was reported previously that among those with HIV, patients that have a psychiatric disorder and use drugs are more nonadherent to their medication than those who only use drugs, or only have a psychiatric disorder [42].

“But that brings up a good point because the concurrent diagnosis usually when you have addictions you have mental health issues, a good chunk of it, and we get that all the time. Mental Health Services will say you deal with your addictions (yeah) and then you come back. And Addictions [Services] says you deal with your mental health.”

In addition, participants observed that PWIDs are difficult to engage. They explained that addiction and the search for the next “fix” often took precedence over any medical or social intervention that the service provider attempted to provide. Some participants highlighted that it is difficult to provide effective services for clients who have no interest in making life changes, or for people who are not able to take primary responsibility for their own self-care. Service providers also discussed the ease with which services “lose” their clients. They stressed that PWIDs frequently lose interest in care if the experience becomes difficult.

“And I think they have good intentions when they leave, they’re talking about . . . new starts and stuff . . . but it’s difficult to . . . if you give them a week or two . . . they’re back to their old behaviours.”

### Successful services

Service providers identified many services in Saskatoon that are useful for PWIDs. The participants recognized characteristics of these successful services that they found helpful or easy to utilize. First and foremost, service providers liked working with agencies whose employees had a good attitude toward PWIDs, attempted to build rapport with their clients, and enjoyed working with PWIDs.

On a more pragmatic note, service providers explained that organizations were especially useful when they were expedient and mobile. The participants noted the value of services that provided PWIDs with material goods, such as blankets and toiletries, and transportation to appointments or between services. They also observed that agencies which assisted with co-ordination of physical needs such as housing and food acquisition were quite helpful for PWIDs.

“They help them with appointments, they help them to figure out food, they help them to look at housing issues . . . and a lot of our clients . . . don’t have that skill, they don’t, they need that extra support to be able to get back to the community.”

The participants appreciated the work that some individuals have done to educate other service providers about the needs of people who inject drugs in the city. They also applauded agencies or individuals which provided a strong advocate voice for PWIDs in the community.

## Discussion

The main purpose of this study was to investigate the experiences of service providers when providing care to PWIDs in Saskatoon. Service providers identified numerous barriers when attempting to provide care to PWIDs. These barriers included stigma and discrimination, inefficient use of resources, and inadequate education. In addition, service providers described PWIDs as a unique and demanding population. Our findings are congruent with the literature regarding some of the barriers identified in accessing care from the perspective of service providers.

Many studies have been conducted that examine the barriers that PWIDs face when accessing services, however, many of them are from the PWIDs’ perspective. To our knowledge, there are a limited number of studies that examine barriers from the perspective of the service provider. Only twelve studies were found in the literature with which we were able to compare our results, ranging in date from 1991 to 2013. Many of these twelve studies have unique goals that make them different from our study and only three have been published since 2008 (Table 1). The lack of research specifically in this area highlights the importance of our study, but has made it difficult to establish if our results correlate with the views of service providers outside of Saskatoon. The details of the search strategy used in our literature searches can be found in Additional file 1.

There were both similarities and differences between the findings of this study and the first phase of the study [14]. A major theme in both phase 1 and phase 2 was discrimination; both PWIDs and service providers felt that stigma and discrimination acted as a barrier to care for PWIDs. Another theme that was found in both studies was a lack of resources and restrictive policies. In phase 1, PWIDs felt a need for shorter waiting lists, greater availability to services and less restrictive policy. These were also identified as barriers by service providers in phase 2. Service providers’ discussed the lack of financial resources that services have, whereas in phase 1, PWIDs discussed the barrier of a lack of personal financial resources . Poor communication with health services was another theme in phase 1 which reflects the education theme found in this study. Both PWIDs and service providers felt that service providers did not know where to send their clients. In phase 1, however, PWIDs described that there is poor communication between services and clients and therefore PWIDs do not know what services are available or where to look for help. Both studies discussed success within the system. PWIDs identified service providers who worked in the area of addictions to be less discriminatory and therefore preferred. Service providers discussed qualities of service providers that made them successful but also described characteristics of services within Saskatoon that are successful. Overall, there were close similarities between the findings of the two complementary studies.

Limitations exist in this study. A small sample size was used and focus groups were not held until no new information arose (saturation of data was not achieved). As previously mentioned, we did not probe for profession specific beliefs as the goal was to understand the beliefs and attitudes of health providers working in addiction services in general. This study served as a good starting point to explore the views of profession specific service providers. Our goal now is to possibly expand this project and perform profession specific investigations to identify their specific beliefs with regards to barriers to accessing care and take into consideration the differences in power between the professions. Also, it was difficult to recruit general practitioners (such as physicians) for the study, as most of the individuals were from addiction services. However, our study participants provided the views of front-line workers who provide services to PWIDs on a regular basis. It is established that increased contact with people who inject drugs leads to less prejudice [20,43] and a specialist group (those who work in clinics or on teams directly aimed at treating people who use drugs) has a more positive attitude toward PWIDs than a non-specialist (general practitioners or physicians) [21]. Therefore, the experiences general practitioners encounter with PWIDs may be different and thus will identify different barriers in providing care.

The focus group format used in this study potentially inhibited self reports of discriminatory behaviour. Service providers may not feel comfortable reporting instances where they have stigmatized PWIDs in an open setting in front of their colleagues. A one-on-one interview may have provided different, more honest results.

### Recommendations

One of the key barriers recognized by service providers was that services are disjointed and difficult to find. Therefore, a suitable solution to the problem may include utilizing a service provider within addiction health services who is familiar with the services within the city, such as a case manager. Case management services provide PWIDs with a case manager who locates and makes services available to the PWID and acts as a mediator between the client and treatment provider [44,45]. PWIDs who are case-managed utilize substance abuse treatment more readily, resulting in reduced alcohol and drug use [44]. There are currently three social workers who are hired as case managers working in Saskatoon. However, these case managers work specifically with people who are HIV positive. Their role is to coordinate health and social care, as well as support and advocate for the client as they navigate through the system. It may be beneficial to strengthen and expand these services in Saskatoon by offering such services beyond the HIV positive population. As such, a more proactive approach, rather than a reactive one, is adopted.

Another possible solution to the lack of knowledge regarding services is to publicize and educate service providers about the resources that already exist. The Sask Street Signs website (http://www.saskstreetsigns.ca) is a resource that allows both service providers and clients to find the name, address, contact information and description of all the services available within Saskatoon. Unfortunately, it is not clear how much this resource is utilized and how often it is updated. Maintaining, promoting and advertising the website as a resource throughout the city (buses, hospitals, shelters) might be useful in informing people of its existence. By improving PWIDs’ and service providers’ knowledge of available resources, better care to PWIDs can be provided.

Service providers could also utilize a program that reduces discrimination through exposure to PWIDs. One program implemented at the University of Saskatchewan allowed medical students to spend a period of four hours with a functioning PWID which greatly improved their perceptions of the PWID. Students reported that patients were regular individuals who have families and personal dreams [data not published]. A report to the Mental Health Commission Canada was done to address the discrimination experienced by those with mental health and addiction problems. In the report the researchers did an extensive review of the literature and found that contact is the most effective single strategy in reducing stigma and discrimination [46]. By developing a program as the one described above, it also allows PWIDs to interact with service providers which may make them more comfortable working with service providers and less likely to perceive discrimination.

One program implemented in Toronto was created to help service providers allow their services to be accessible and supportive to people living with concurrent mental health and substance abuse [47]. The program provided mental health and addiction workers with tools to use to raise awareness about the stigma associated with concurrent disorders. This program titled 'Beyond the Label’ is an educational kit that includes ten group activities, background information on concurrent disorders and stigma, discussion points for the group and examples of times to use the kit in the community. It is possible that such a tool kit could be used by service providers in Saskatoon or a similar urban setting to address the issue of discrimination as a barrier to providing care and improving the accessibility of services to PWIDs [47].

The ultimate goal of the above recommendations is to be able to provide optimum care to PWIDs and as a result to reduce some of the complications associated with injection drug use including infections, psychiatric illness and death due to overdose. These recommendations will improve PWIDs access to care in Saskatoon. We, however, recognize that longer term solutions will be required for optimizing care such as addressing issues surrounding waiting lists, staffing/workload, restrictive rules and regulations, and appropriate use of emergency rooms, and education of service providers. We are currently investigating the educational needs, concerning substance abuse, for pharmacists within Saskatchewan, Canada.

## Conclusion

In summary, service providers have recognized barriers that are preventing optimal care to PWIDs during a crisis situation. A previous phase of this study, in which PWIDs identified the barriers that they felt were present when accessing services, has been completed and results have been compiled. The next step that must be executed is to communicate the information established in these studies to stakeholders in order to implement new policies/initiatives within the Saskatoon Health Region. Community organizations and health services in Saskatoon can now benefit from our findings. Recommendations for new initiatives include expanding the cliental of case managers, and providing training programs to service providers to improve their level of knowledge and skills regarding people who inject drugs. By implementing such recommendations, people who inject drugs may have better access to services and improved care, leading to better quality of life and a reduction in co morbidities among PWIDs in Saskatoon and other small urban centers.

## Abbreviations

PWID: People who inject drugs; HIV: Human immunodeficiency virus; HCV: Hepatitis C virus.

## Competing interests

The authors declare that they have no competing interests.

## Authors’ contributions

KL Designed the questions for the study; conducted some focus groups; trained J. Neil in focus group data analysis; gathered literature; performed data analysis; prepared ethics application; wrote parts of the manuscript. JN: Gathered literature; performed data analysis; drafted most of the manuscript. JW: Designed questions for the study; recruited participants; provided significant input into recommendations. CAD: Assisted in the preparation of the ethics application; co- designed the study; provided input into data analysis. SB: Trained K. Lang in focus group data analysis; provided input into data analysis. AEA: Principle investigator, designed the study, trained focus group facilitators, recruited participants, conducted some focus groups; prepared ethics application; performed data analysis; revised subsequent manuscript drafts. All authors read and approved the final manuscript.

## Supplementary Material

Additional file 1 Search Strategy. Click here for file
