# Supplementary material for: Qualitative investigation of barriers to accessing care by people who inject drugs in Saskatoon, Canada: perspectives of service providers
Source: Subst Abuse Treat Prev Policy. 2013 Oct 1;8:35. doi: 10.1186/1747-597X-8-35 (PMC3850796; doi:10.1186/1747-597X-8-35)
Supplement: Additional file 1 — Search Strategy. [file 1747-597X-8-35-S1.docx]

**Appendix A: Search Strategy**

Six databases were searched: Medline, Embase, Proquest Public Health, PubMed, PsycInfo and Google Scholar. Medline, Embase, PubMed and Proquest Public Health were searched for their coverage of health issues and service providers; PsycInfo searched for its focus on psychology including that of service providers; and Google Scholar for its wide coverage of many areas of literature. The Medline search included publications from 1946 – 2012 and was completed on February 29, 2012; Proquest from 1972 – 2012 and completed on February 29, 2012; Embase from 1947 – 2012 and completed on April 20, 2012; PsycInfo from 1806 – 2012 and completed on April 20, 2012; PubMed from 1966 – 2012 and completed on April 20, 2012; Google Scholar on February 29, 2012.

Each search used a combination of subject headings and keywords. The following search strategies were used for each database.

**Medline**

1. exp Health Occupations/
2. exp Allied Health Occupations/
3. exp physician assistants/
4. "service provider*".mp. [mp=title, abstract, original title, name of substance word, subject heading word, protocol supplementary concept, rare disease supplementary concept, unique identifier]
5. 1 or 2 or 3 or 4
6. exp substance abuse, intravenous/
7. exp *Substance-Related Disorders/
8. 6 or 7
9. *"Attitude of Health Personnel"/
10. exp delivery of health care/
11. 5 and 8 and 9 and 10 (537 results)
12. limit 11 to (English language and humans) (496 results)

**Proquest Public Health**

1. Exp Health Occupations
2. Exp Allied Health Occupations
3. 1 or 2
4. Exp Substance Abuse, Intravenous
5. Exp Delivery of Health Care
6. Health Services Accessibility
7. 5 or 6
8. 3 and 4 and 7 (190 results)
9. Attitude of Health Personnel
10. 7 or 9 (105 results)
11. 8 or 10 (208 results)

**Embase**

1. Exp Health personnel
2. Exp Physician Assistants
3. Exp Allied Health Personnel
4. Service provider.mp
5. 1 or 2 or 3 or 4
6. Health Personnel attitude
7. Exp Health care delivery
8. 6 or 7
9. Exp substance abuse, Intravenous
10. 5 and 8 and 10 (97 results)
11. Limit to English language and humans (90 results)

**Pubmed**

1. Health Professional
2. Service provider
3. Service provider attitudes
4. 1 or 2 or 3
5. Substance abuse
6. Intravenous drug abuse
7. 5 or 6
8. Attitude of health personnel
9. 4 AND 7 AND 8 (8 results)

**PsycInfo**

1. Exp Health Personnel
2. Exp Allied Health Personnel
3. Service provider.mp
4. 1 or 2 or 3
5. Exp intravenous drug usage
6. Exp Health Personnel attitudes
7. Exp Health Care Delivery
8. Exp Treatment Barriers
9. 6 or 7 or 8
10. 4 and 5 and 9 (14 results)
11. Limit to human and English language (14 results)

A total of 816 studies were found using these strategies, of which 8 met the inclusion criteria. The results were carefully analyzed to find comparable studies based on the title, abstract and, if necessary, the full article. Studies that examined barriers that injection drug users face when accessing care were saved to be further analyzed. Studies that did not involve injection drug users were excluded as well as those not from the perspective of service providers. A variety of services were included, including harm reduction services and methadone clinics.
